# Population Pharmacokinetic Modeling of a Desmopressin Oral Lyophilisate in Growing Piglets as a Model for the Pediatric Population

**DOI:** 10.3389/fphar.2018.00041

**Published:** 2018-01-31

**Authors:** Elke Gasthuys, An Vermeulen, Siska Croubels, Joske Millecam, Stijn Schauvliege, Thomas van Bergen, Pauline De Bruyne, Johan Vande Walle, Mathias Devreese

**Affiliations:** ^1^Department of Pharmacology, Toxicology and Biochemistry, Ghent University, Merelbeke, Belgium; ^2^Department of Bio-analysis, Faculty of Pharmaceutical Sciences, Ghent University, Ghent, Belgium; ^3^Department of Surgery and Anesthesiology of Domestic Animals, Faculty of Veterinary Medicine, Ghent University, Merelbeke, Belgium; ^4^Department of Pediatrics and Medical Genetics, Faculty of Medicine and Health Sciences, Ghent University, Ghent, Belgium

**Keywords:** piglet, animal model, pharmacokinetics, desmopressin, pediatric

## Abstract

Desmopressin is used to treat primary nocturnal enuresis in children. Over the years, various formulations of desmopressin were commercialized of which the sublingual melt tablet is preferred in the pediatric population, despite the lack of full PK studies in this population. A full PK study was performed in growing conventional piglets to evaluate if this juvenile animal model can provide supplementary information to complement the information gap in the pediatric population. A desmopressin sublingual melt tablet (120 μg) was administered to 32 male piglets aged 8 days, 4 weeks, 7 weeks, and 6 months (each group *n* = 8). Population PK (pop-PK) analysis was performed to derive the PK parameters, the between- and within-subject variabilities and the effects of covariates. Desmopressin demonstrated two-compartmental PK, with a dual, sequential absorption process, and linear elimination. Body weight was the only significant covariate on clearance and on apparent volume of distribution of the central compartment. In human pediatric trials, no double peak in the absorption phase was observed in the plasma concentration-time curves, possibly due to the sparse sampling strategy applied in those pediatric studies. Therefore, it is recommended to perform additional studies, based on the sampling protocol applied in the current study.

## Introduction

Pediatric non-clinical and clinical studies are pivotal to investigate *inter alia* age-dependent dosing schedules and age-appropriate formulations. In order to stimulate the industry to perform such pediatric clinical trials, the FDA (Food and Drug Administration) and EMA (European Medicines Agency) issued several regulations, such as the US Best Pharmaceuticals for Children Act (2002), Pediatric Research Equity Act (2003) and the Pediatric Regulation (2006). Despite the increased regulatory efforts, there is still a lack of clinical trials in children, and consequently a lack of medicines labeled for children (±50% of the drugs are prescribed off-label).

**Figure 1:**
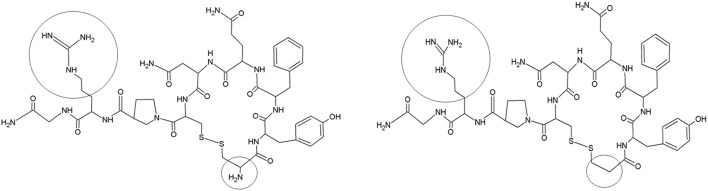
Structure of vasopressin (left) and desmopressin (right). Structural differences are marked (black circle).

Desmopressin (1-desamino-8-D-arginine vasopressin, Figure 1) has an antidiuretic effect through binding on the vasopressin 2 (V_2_) receptors of the kidney collecting ducts. It was initially developed for the indication of central diabetes insipidus in adults, but was subsequently used to treat primary nocturnal enuresis (PNE) in children (Cobb et al., 1978; Rittig et al., 1989; Glazener and Evans, 2002). Over more than 30 years, two oral [tablet (1987) and lyophilisate (2005)] and one nasal formulation [spray (1972)] have been developed to reduce PNE in children. In 2007, the use of intranasal formulations was no longer approved for treating PNE in children in the USA and most of the European countries, because of reported cases of severe hyponatremia and seizures. Therefore, a switch to oral formulations was required, despite the limited number of data on the PK of oral desmopressin in children, the lack of bioequivalence studies between the tablet and the oral lyophilisate, and the lack of availability of size-dependent dosing regimens in the pediatric population. Osterberg et al. (2006) performed a pharmacokinetic (PK)/pharmacodynamic (PD) study (oral lyophilisate, 30, 60, 120, 360, or 480 μg) in 84 enuretic children aged 6–12 years and applied a sparse sampling protocol (2 to 3 blood samples/child). Also De Bruyne et al. (2014) applied a sparse sampling protocol after administration of conventional tablets (200 μg) and oral lyophilisates (120 μg) containing desmopressin to 23 children (5–18 years) suffering from monosymptomatic nocturnal enuresis. Blood was sampled at 1, 2, and 6 h post administration. The full PK profiles were obtained through pop-PK simulation (Michelet et al., 2016), but likely do not capture all characteristics of desmopressin's PK, since the original studies applied a sparse sampling protocol. It is in this respect, that trials using pediatric animal models, such as the conventional piglet, might provide supplementary information to complement the information gap since full PK studies can be performed.

The overall aim of the present study was to evaluate whether the conventional piglet is a potentially relevant animal model for PK studies of desmopressin. The objective was to adequately describe the desmopressin plasma concentration-time profiles in piglets of 8 days, 4 weeks, 7 weeks, and 6 months, using non-linear mixed effects modeling (NLME). These age groups correspond to the main age categories of the human pediatric population, namely neonate, infant, child, and adolescent (Gad, 2007; Gasthuys et al., 2016). The estimated values of the primary PK parameters correlated with the values obtained in children, also obtained using pop-PK analysis.

## Materials and methods

### Animals

The current study was approved by the ethical committee of the Faculties of Veterinary Medicine and Bioscience Engineering of Ghent University (EC2014/184). Care and use of animals was in full compliance with the national (Belgian Royal Decree, 2013) and European legislation (Anonymous, 2010). A total of 32 stress-resistant male piglets (Landrace × large white, Seghers Hybrid®, Wuustwezel, Belgium) aged 8 days (*n* = 8, 2.01 ± 0.43 kg), 4 weeks (*n* = 8, 10.0 ± 1.69 kg), 7 weeks (*n* = 8, 13.9 ± 2.74 kg), and 6 months (*n* = 8, 112.88 ± 9.11 kg) were used. The animals were group housed in rescue decks (8 days) (0.9 × 1.40 m, Provimi, Rotterdam, The Netherlands) and standard pig stables (4 and 7 weeks, 6 months) (2.30 × 2.40 m), and had *ad libitum* access to feed (8 days: RescueMilk®, Provimi; 4 and 7 weeks: Piggistart Opti®, 6 months: Optivo Pro®, Aveve, Leuven, Belgium) and drinking water. Natural light was provided by translucent windows and the stable temperature was 23.2 ± 0.56°C. Higher temperatures were obtained in the rescue decks through heating lamps. In all age groups, a double lumen catheter was placed in the jugular vein according to Gasthuys et al. (2017b). After surgery, the piglets were housed individually to avoid displacement of the catheters. During single housing, a cotton towel was added to the decks and stables to mimic the smell of the other piglets. During the experiments, the stables were enriched with hanging chains, rubber toys and balls of different sizes. The environmental enrichment was rotated daily.

### Experimental design

After one acclimatization day, a desmopressin oral lyophilisate (Minirin® Melt 120 μg, kindly donated by Ferring, Copenhagen, Denmark) was placed under the tongue of all the piglets. Afterwards, the snout was closed during 30 s to ensure full dissolution of the lyophilisate. The animals were deprived of milk or feed 1 h before and 1.5 h after administration of desmopressin. Venous blood samples (1 mL) were collected through the distal lumen of the catheter at 0 (just prior to administration), 5, 15, 30, 60, min and 1.5, 2, 3, 4, 6, 8, 10, 12 and 24 h (post administration). The samples were drawn into 9 mL EDTA-K_3_ collection tubes (Vacutest®, Piove di Sacco, Kima, Italy), immediately kept on ice and centrifuged for 10 min at 2,095 × g within 2 h after blood collection. The plasma was aliquoted, frozen and stored at −80°C until analysis (within 2 months). The body surface of the piglets (8 days, 4 and 7 weeks) was imaged using a four-slice helical CT-scanner (Lightspeed QC/I, GE Medical systems, USA, New York). The CT-scans were processed using VGStudioMax (Volume Graphics GmbH, Heidelberg, Germany) and the body surface area (BSA) was determined after visualization and selection of the body surface in 3D (UGCT-Department of Physics and Astronomy, Ghent University, Ghent, Belgium). The GFR was determined as the clearance of the exogenous marker exo-iohexol (Gasthuys et al., 2017a). After the experiment, euthanasia was performed by administering an overdose of sodium pentobarbital (sodium pentobarbital 20%®, Kela, Belgium) through the proximal lumen of the jugular catheter.

### Desmopressin analysis

Desmopressin plasma concentrations were determined at Aarhus University Hospital (Aarhus, Denmark) using a validated radioimmunoassay method as described by Emmeluth et al. (1994) and Bie and Sandgaard (2000). A 1-desamino-8-D-arginine vasopressin antibody (C990728, Biogenesis Ltd., Poole, UK) was used at a final dilution of 1:125,000. The limit of quantification (LOQ) was 4.2 pg/mL and the intra- and inter-assay coefficient of variation was 7.9and 12.1%, respectively.

### Pharmacokinetic analysis

#### Software

The data and statistical analysis comply with the recommendations on experimental design and analysis of pharmacology (Curtis et al., 2015). Model development and parameter estimation were performed by non-linear mixed effect modeling using NONMEM® version 7.3. (Icon Development Solutions, Ellicott City, MD, USA).

#### Structural model development

The first-order conditional estimation method was used as the estimation algorithm for all analyses. Plasma concentrations below the LOQ were excluded from the analysis (4 pg/mL; *n* = 8%) and a logarithmic transformation of the remaining plasma concentrations was performed. A one-compartmental model with first-order absorption was chosen as the pop-PK base model, analogous to the human pop-PK model development (Michelet et al., 2016). Subsequently, based on a visual exploration of the plasma concentration-time profiles obtained in the growing piglets, a two-compartmental disposition model and a dual-input were evaluated to describe the absorption of the sublingual desmopressin lyophilisate. Various input functions were additionally explored, for an optimal fitting of the observed input profile. Structural model selection was guided by visual inspection of goodness-of-fit plots, the objective function value (OFV), Akaike's information criterion (AIC) and the condition number (CN). The models were chosen based on mechanistic plausibility and smaller values of OFV and/or AIC and acceptable CN.

### Stochastic model development

The inter-individual variability (IIV) on clearance, volume of distribution and the absorption parameters was assumed to be log-normally distributed and was modeled using an exponential-error model. The residual variability was described using the log-transform both sides approach, which implies that the log of the concentrations was modeled with an additive error (proportional error model in the untransformed domain).

#### Covariate model development

Once the structural and error models were obtained, continuous covariates were included using the stepwise covariate modeling approach (forward selection followed by backward elimination). Evaluated continuous covariates on central volume of distribution (V_1_/F), apparent total body clearance (CL/F) and absorption-related parameters were body weight (BW), BSA, age and/or GFR. A covariate was considered significant when its addition resulted in a decrease in OFV of more than 6.635 (χ^2^ test, *df* = 1, *p* < 0.01) and 10.828 (χ^2^ test, *df* = 1, *p* < 0.001) after forward inclusion and backward elimination of one covariate, respectively.

#### Model evaluation

The final model was evaluated based on diagnostic plots of the individual (IPRED) and population (PRED) predicted concentrations vs. the observed concentrations (C_obs_), and time after dose and PRED vs. the conditional weighted residuals (CWRES). Evaluation of the diagnostic plots was based on visual inspection of the plots for scatter and bias. The precision of the parameter estimates was determined by calculating the relative standard error (%RSE). The fixed- and random-effect estimates with relative precision below 30% and 40–50%, respectively, were considered as precisely estimated. A visual predictive check (VPC) was performed to evaluate the predictive properties of the final model based on 1,000 simulations of the original dataset. The dependent variable C_obs_ (y-axis) was plotted against the independent variable time after dosing (x-axis). The 50th percentile (median) of the observed data and the simulated predictions were presented. Moreover, the 5th (lower) and 95th (upper) percentiles were depicted, corresponding to an inter-percentile range of 90% (= prediction interval). For all simulated percentiles, the 95% confidence interval was also presented.

### Secondary PK parameters

The individual PK parameter estimates (= empirical Bayes estimates, EBE) were computed during the estimation of the final population parameters. The shrinkage was determined to evaluate the quality of the EBE estimation, since the EBE tends to shrink toward the population mean in case of non-informative data. Shrinkage below 30% was considered acceptable. The secondary parameters, namely AUC_0 → ∞_ and T_½el_ were calculated. The individual AUC values were determined by the logarithmic trapezoidal method with extrapolation to infinity.

### Statistical analysis

The effect of age on AUC_0 → ∞_ and T_½el_ was analyzed using one-way analysis of variance after determination of normality and variance homogeneity (SPSS 24, IBM, United States).

## Results

### Population characteristics

All piglets survived catheterization of the jugular vein without any complications. All piglets received a desmopressin oral lyophilisate under identical circumstances (deprived from food, properly hydrated) and no general side effects were observed during the experiment. The descriptive (mean ± SD and median [range]) characteristics are detailed in Table 1.

**Table 1:** Descriptive characteristics of the piglet study population.

**Characteristic**		**8 days**	**4 weeks**	**7 weeks**	**6 months**
N° of piglets		8	8	8	8
BW (kg)	Mean Median	2.01 ± 0.43 2.21 [1.54–2.64]	10.0 ± 1.69 10.0 [7.0–12.0]	15.8 ± 1.98 15.0 [14.0–19.0]	112.9 ± 9.11 113.6 [100.0–124.0]
BSA (m^2^)	Mean Median	0.16 ± 0.02 0.17 [0.13–0.19]	0.46 ± 0.05 0.46 [0.36–0.52]	0.59 ± 0.08 0.63 [0.60–0.73]	2.42 ± 0.13 2.43 [2.23–2.58]
GFR (mL/min/m^2^)	Mean Median	40.8 ± 8.62 40.6 [24.5–50.5]	67.4 ± 11.54 66.2 [54.2–82.2]	106.5 ± 18.69 110.7 [77.8–133.9]	136.3 ± 13.48 135.5 [120.2–159.0]

*Values are presented as mean ± SD and median [range]. N°, number; BW, body weight; BSA, body surface area; GFR, glomerular filtration rate adapted from Gasthuys et al. (2017b)*.

### Pharmacokinetic analysis

**Figure 2:**
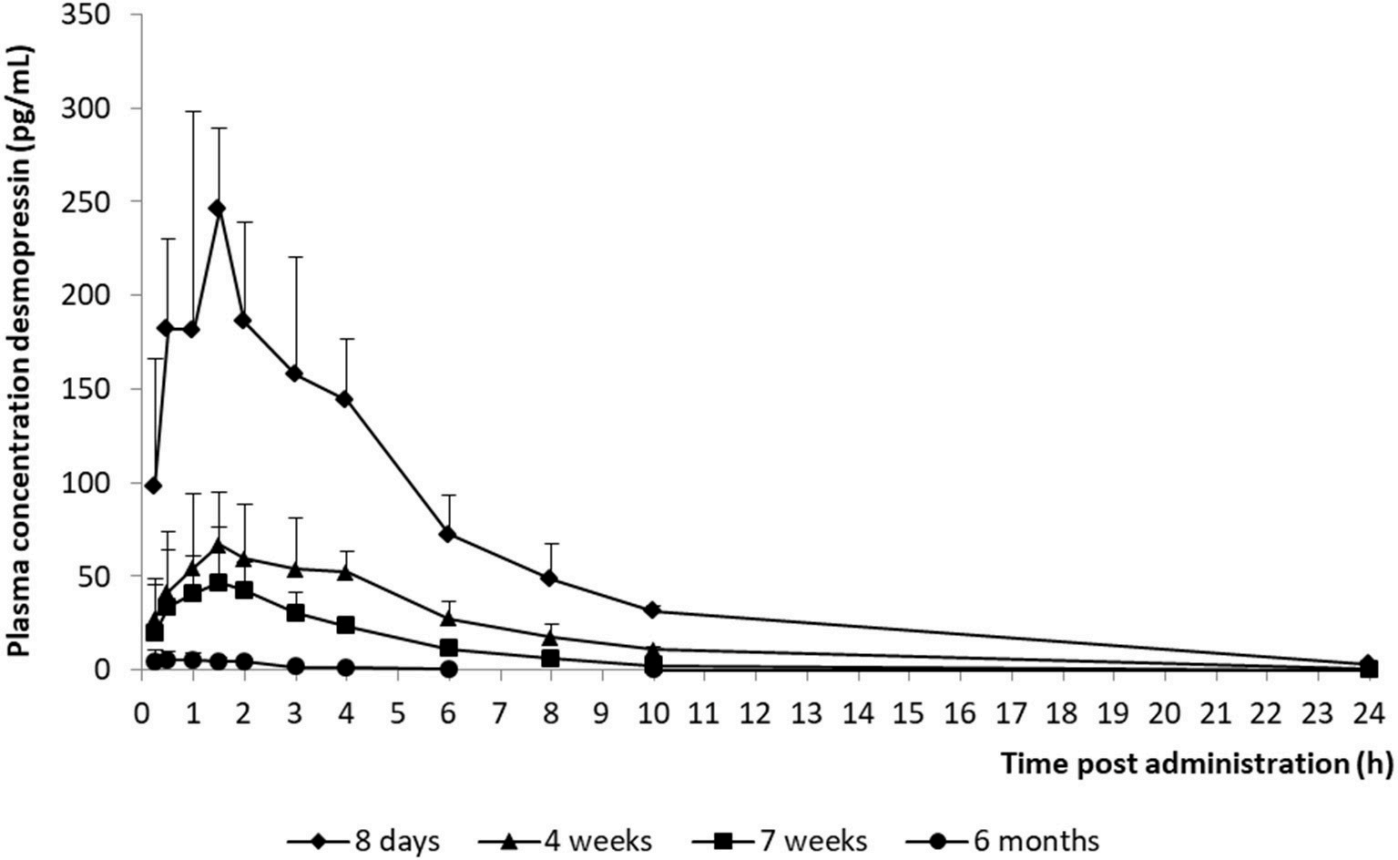
Mean (+ SD) desmopressin plasma concentration vs. time curves of piglets aged 8 days (*n* = 8), 4 weeks (*n* = 8), 7 weeks (*n* = 8), and 6 months (*n* = 8) after receiving a desmopressin oral lyophilisate (120 μg).

**Figure 3:**
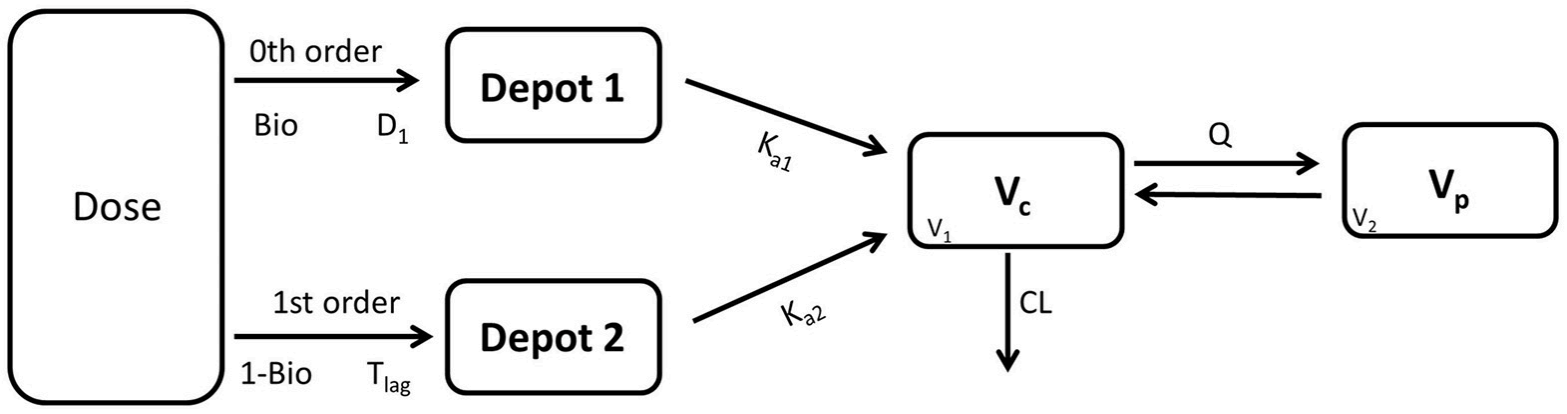
Graphical representation of the applied model to describe the pharmacokinetics of a desmopressin oral lyophilisate in growing piglets. D_1_: duration 1, T_lag_: lag time, Bio: fraction of the absorbed dose (buccal absorption), 1-Bio: fraction of the absorbed dose (assumed gastro-intestinal absorption), K_a1_/K_a2_: first-order absorption rate constants, V_c_(V_1_): apparent volume of distribution of the central compartment, V_P_(V_2_): apparent volume of distribution of the peripheral compartment, Q: inter-compartmental flow, CL: apparent total body clearance.

**Figure 4:**
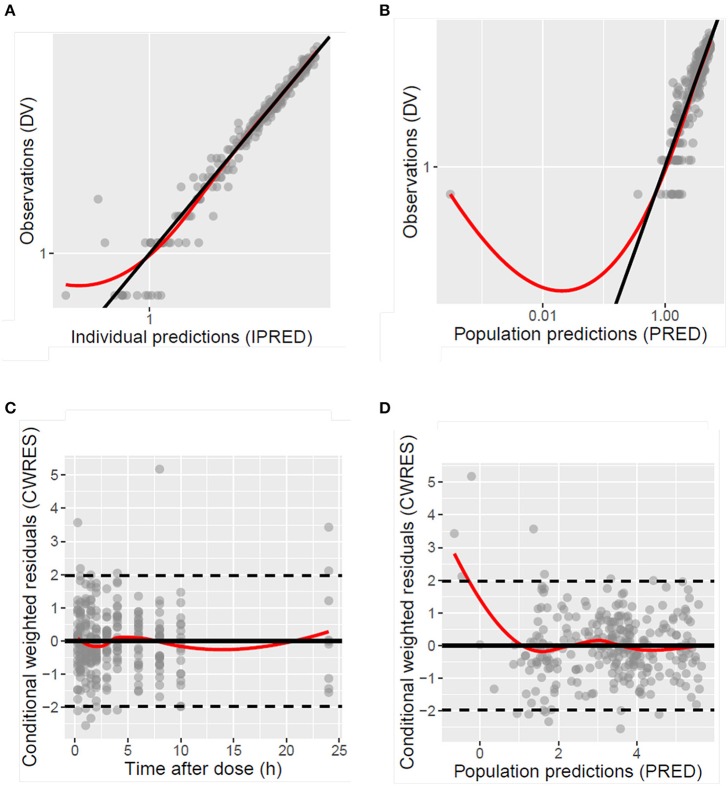
Diagnostic plots of the population model of desmopressin after oral lyophilisate administration to growing piglets: scatter plots of the dependent variable (DV), namely observed plasma concentrations (C_obs_), vs. the individually predicted plasma concentration values (IPRED) **(A)** and population predicted plasma concentration values (PRED) **(B)**, scatter plots of the conditional weighted residuals (CWRES) vs. time after dose **(C)** and PRED **(D)**. Gray circles represent the observed data, the full black lines are the lines of unity and the full red lines are the LOESS smoothed lines.

**Figure 5:**
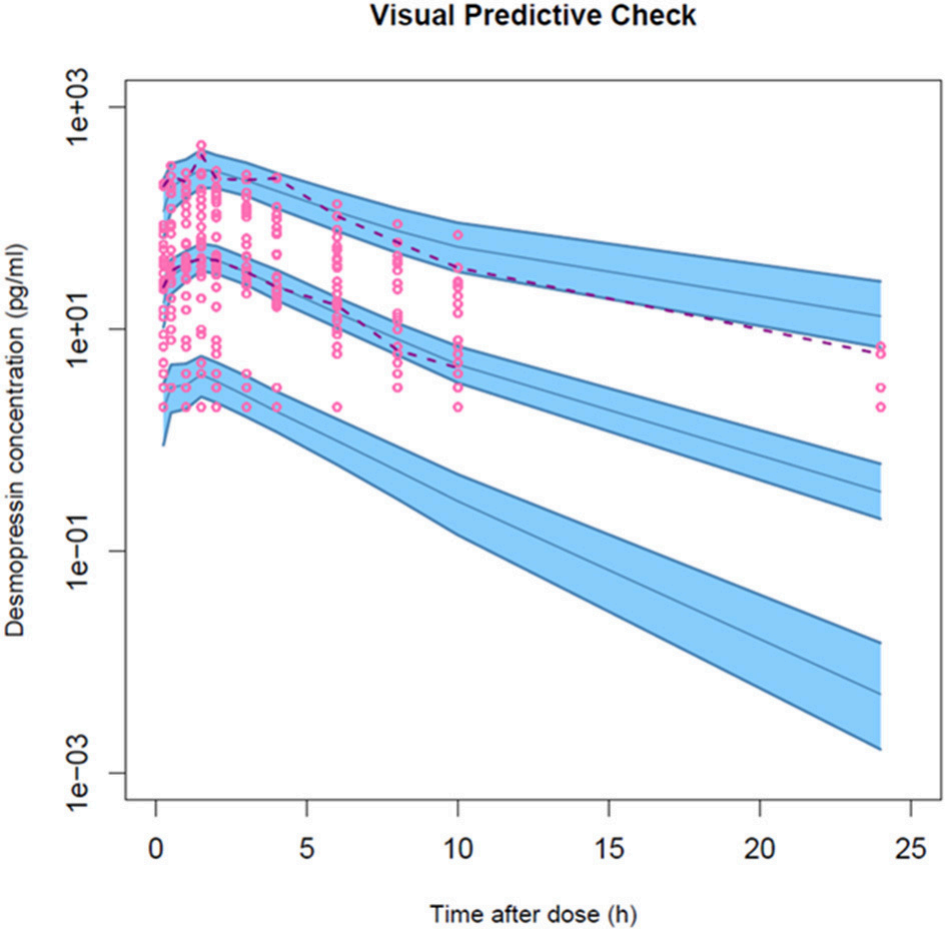
Visual predictive check for the final population PK model of a desmopressin oral lyophilisate. Gray circles represent the observed data. The lower and upper dashed lines and solid line represent the 5th, 95th, and 50th percentiles, respectively. The shaded areas are the 95% confidence interval of the corresponding percentiles.

The model development path is described in Supplementary Table 1. Figure 2 shows the plasma concentration-time profiles of desmopressin. The best description of the plasma concentration-time profiles of desmopressin in growing piglets was obtained with a two-compartmental disposition model combined with a dual input function for the absorption. A zero-order release into a first depot followed by first-order absorption, and a first-order release from a second depot after a lag time (T_lag_), both into the central compartment was chosen as the final model (Figure 3). This model was required since many plasma concentration-time profiles showed a 2nd peak in their profile implying that most probably, a part of the dose has been ingested (1-Bio) rather than directly absorbed through the buccal mucosae (Bio). The main parameters included in the model were apparent total body clearance (CL/F), apparent central and peripheral volume of distribution (V_1_/F; V_2_/F), apparent intercompartmental flow (Q/F), first-order absorption rate constants from the first and second depot (Ka_1_; Ka_2_), the fraction of the dose split over depot 1 and depot 2 (Bio), the lag time (T_lag_) and duration of the zero-order release (D_1_). Since no intravenous data were obtained in piglets, the absolute oral bioavailability (F) could not be estimated. The IIV of Q, Ka_2_ and T_lag_ could not be estimated from the data, and was fixed to zero. The forward inclusion and backward elimination of the covariate effect BW on apparent clearance (CL/F) and apparent central volume of distribution (V_1_/F) demonstrated a significant improvement of the model (ΔOFV = 14.43 and 39.72, respectively). No other covariates, including renal function and age, were found to be significant for any of the parameters. The final pop-PK model parameter estimates are presented in Table 2. Model diagnostic plots presented in Figure 4 show the overall goodness-of-fit of the final model. The IPRED (Figure 4A) vs. C_obs_ and PRED (Figure 4B) vs. C_obs_ plots demonstrated no significant deviations of the LOESS smoothed line from the line of unity, meaning that appropriate structural models could be fitted for most individuals and no major bias in the population components of the model were found. The scatter plot of the CWRES vs. time (Figure 4C) showed a symmetric distribution of the weighted residuals around zero across the entire range of time after dosing, implying that no major bias was observed. The scatter plot of the CWRES vs. PRED (Figure 4D) showed positive WRES in the lowest PRED, indicating a slight overprediction of the lowest concentration values. The %RSE of the fixed- and random-effect parameters were regarded acceptable [even though for V_2_/F, it was just above 30% (31.4%)]. Most of the observations are within the 90% prediction interval of the VPC (Figure 5) indicating that the model could reliably predict the observations indicative of a good performance of the final model.

**Table 2:** Population pharmacokinetic model parameter estimates after administration of a desmopressin oral lyophilisate (120 μg) to growing piglets. Body weight is centered on the typical weight of 10 kg.

**Parameter**	**Estimate (% shrinkage)**	**SE**	**RSE (%)**
**STRUCTURAL MODEL**			
CL/F = θ^1^ × (BW/10)^θ11^ × e^η1^	395 L/h	31.8	8.05
V_1_/F = θ^2^ × (BW/10)^θ12^ × e^η2^	131 L	21.1	16.1
Ka_1_ = θ^3^ × e^η3^	0.275 h^−1^	0.0272	9.89
Q/F = θ^4^	32 L/h	6.8	21.3
V_2_/F = θ^5^ × e^η5^	436 L	137	31.4
D_1_ = θ^6^ × e^η6^	0.16 h	0.0473	29.6
Ka_2_ = θ^7^	0.399 h^−1^	0.0677	17.0
Bio = θ^8^	86%	0.0488	5.67
T_lag_ = θ^9^	1 h	0.00196	0.20
**COVARIATE MODEL**			
Influence of BW on CL	1.03	0.0627	6.09
Influence of BW on V_1_	0.691	0.135	19.54
**INTER-INDIVIDUAL VARIABILITY**			
IIV CL/F	0.175 (1.97%)	0.0462	13.20
IIV V_1_/F	0.641 (25.4%)	0.264	20.59
IIV Q/F	0 (FIX)	0	0
IIV Ka_1_	0.0903 (16.0%)	0.0339	18.77
IIV Ka_2_	0 (FIX)	0	0
IIV V_2_/F	0.634 (61.8%)	0.202	15.93
IIV D_1_	0.485 (47.4%)	0.255	26.29
IIV Bio	0.627 (42.9%)	0.391	31.18
IIV T_lag_	0 (FIX)	0	0
**RESIDUAL ERROR**			
ADD = θ^10^	22.8% CV	0.0291	12.76

*CL/F, apparent total clearance after oral administration; V/F, apparent central (V_1_/F) and peripheral (V_2_/F) volume of distribution after oral administration; Ka_1_/Ka_2_, first-order absorption rate constants from depot 1 and 2, respectively; Q/F, apparent intercompartmental flow; D_1_, duration of the zero-order input, Bio: fraction passing through depot 1; T_lag_, lag time depot 2; BW, body weight; IIV, inter-individual variability; ADD, additive residual error; CV, coefficient of variation; SE, standard error; RSE, residual standard error*.

The individual primary PK parameters were obtained directly from the model, through the posthoc step (Bayesian feedback step). The shrinkage of the primary parameters was acceptable (Table 2). The shrinkage of V_2_/F was above 30%, but since the shrinkage applies to the complete dataset, the values were still used to calculate the V_SS_. The values for the secondary PK parameters, AUC and T_½_ are depicted in Table 3. No significant differences in AUC and T½ (*p* < 0.05) between the 4- and 7-week-old piglets were observed. A significant difference in AUC and T½ was observed between the 6-month-old piglets and the other age groups. Moreover, a significant difference in AUC and T½ was observed between the 8-day-old piglets and the other age groups except between the T½ of the 8-day-old and 4-week-old piglets.

**Table 3:** Mean (±SD) secondary PK parameter estimates after administration of a desmopressin oral lyophilisate (120 μg) in growing piglets at 8 days, 4 weeks, 7 weeks and 6 months.

**Parameter**	**8 days**	**4 weeks**	**7 weeks**	**6 months**
AUC_0 → ∞_ (pg^*^h/mL)	1369 ± 485^a,b,c^	436 ± 196^a,d^	215 ± 94.1^b,e^	32.7 ± 19.1^c,d,e^
T_½el_ (h)	3.83 ± 2.56^f,g^	1.40 ± 0.58^h^	0.90 ± 0.32^f,i^	0.10 ± 0.04^g,h,i^

*AUC area under the plasma concentration-time curve, T_1/2el_ half-life. Results with the same numerical character superscript are considered significant different (p < 0.05)*.

## Discussion

PK studies in the pediatric population are essential to determine correct dosage strategies for optimal efficacy. Despite worldwide use of desmopressin to treat nocturnal enuresis in children, full PK studies taking growth and maturation into account are still lacking. To date, most studies investigating the PK of desmopressin in this population applied a sparse sampling protocol on a limited number of patients (Osterberg et al., 2006; De Bruyne et al., 2014). Informative animal models, such as the conventional piglet, can be used to perform full PK studies to provide additional information such as sampling strategies for human pediatric clinical trials.

In the present study, a desmopressin oral lyophilisate (120 μg) was administered to growing piglets of 8 days, 4 weeks, 7 weeks, and 6 months old. A two-compartmental pop-PK model with absorption described by a dual input function best fitted the data. Such a dual input function was required to describe the double peak phenomenon in the plasma concentration-time curves. The first input function comprises a zero-order absorption rate, most probably related to buccal absorption of the oral lyophilisate. No lag time was required for the first input function, indicating that sublingual absorption occurred rapidly after oral administration of the oral lyophilisate. The second input function comprises a first-order absorption rate into the systemic circulation, possibly related to gastro-intestinal absorption after swallowing part of the dose. A lag time of 1 h was modeled for the second input function, indicating that the gastro-intestinal absorption did not start immediately after oral administration of the oral lyophilisate. A double peak has not been observed in the plasma concentration-time curves of a desmopressin oral lyophilisate in pediatric clinical PK studies. Two possible explanations can be given, first, the piglet has a different absorption of desmopressin in comparison with humans. Second, the double peak might be missed in the human studies due to the sparse sampling design applied in the pediatric studies. Consequently, additional studies with more intensive sampling in the absorption phase are required to determine whether or not the piglet is a suitable model to study the PK of desmopressin. This conclusion is in accordance with the conclusions drawn by Michelet et al. (2018), who found a significant effect of formulation (oral vs. melt) on the IC_50_. This effect is physiologically implausible, since a formulation effect is expected at the PK-side rather than the PD-side. A possible explanation given by the authors was the sparser sampling protocol applied in the pediatric human studies. The absorption of desmopressin sublingual melt tablet might appear rapidly, potentially missing a larger peak or a second peak in the concentration-time curve. An adaption of the sampling strategy in the pediatric clinical trials is advised with thorough sampling during the absorption phase.

Most articles describing the PK parameters of desmopressin administered to adults (Fjellestad-Paulsen et al., 1993; Agersø et al., 2004; Fransén et al., 2009) applied a two- or three-compartmental model to fit the data. Michelet et al. (2016) combined the data collected in children by Osterberg et al. (2006) and De Bruyne et al. (2014) and concluded that a one-compartmental model with first-order absorption best described the data. In the current study, a two-compartmental model was chosen based on a smaller OFV (ΔOFV = 87.42) in comparison with a one-compartmental model, despite the high values of RSE and shrinkage for V_2_/F (31.4 and 61.8%, respectively). Since our experimental design comprised a rich sampling protocol, including a 24-h time point and younger age groups, it is possible that these factors favor a two-compartmental model, which was also fitted to the adult human data.

The apparent CL/F population value is 395 L/h. BW was a significant covariate, suggesting that individuals with higher BW have a higher absolute clearance. Since renal excretion is the main route of elimination of desmopressin (Fjellestad-Paulsen et al., 1993) and catabolism of desmopressin appears after active uptake by the proximal tubules of the kidney (Maack et al., 1979), kidney maturation might thus explain the increased clearance with increasing BW. When including the covariate GFR, a significant improvement of the OFV was observed, but the covariate was not retained, since backward elimination did not reach the pre-specified significance level. Consequently, maturation of the GFR could only partially explain the increase in clearance. When looking at the data, it should be noted that the apparent clearance is presented in the current study, since the F in piglets is not known. Therefore, the age-related difference in clearance might also be attributable to a significant difference in F as well. Since the F in humans is very low (< 1%), this might also be the case in piglets. Consequently, when considering the F in growing piglets, a small difference in F could potentially lead to a significant difference in clearance. Therefore, a follow-up study should be performed, to determine the absolute oral bioavailability in growing piglets. In humans, the mean population value of CL/F after oral dosing of desmopressin in a fasted population of children was 2330 L/h (Osterberg et al., 2006) and 2098 L/h (Michelet et al., 2016). No significant effect of BW on CL/F could be observed in both studies. This might be explained by the age ranges, Osterberg et al. (2006): 6–12 years (Child), De Bruyne et al. (2014): 5–18 years(child-adolescent), and consequently the BW ranges included in those studies that were smaller than in the current study (neonate-adolescent), whereby the covariate effect of BW on clearance might be underestimated. A good correlation can be found between the values obtained by Michelet et al. (2016) and the values obtained by allometric scaling, namely CL (human) = CL (pig)^*^(BW human/BW pig)^0.75^ (ratio: 1.70), rendering the piglet a good animal model to predict the clearance of desmopressin in humans.

The apparent V_1_ population estimate is 131 L. As seen with clearance, a significant covariate effect of BW was found, suggesting that individuals with higher BW have also an increased volume of distribution of the central compartment. Since desmopressin has a negative clog P (−7.711), demonstrating the hydrophilicity of this drug, the maturational changes of the body composition could explain the covariate effect of BW on V_1_ (Lawton and Witty, 2013). However, it should be noted that the V_1_ is also the apparent volume of distribution, so the age-related difference could again be due to significant differences in F. Therefore, also here, follow-up studies should be performed, to determine the F in growing piglets. In humans, the mean population value of V_d_/F after oral dosing of desmopressin in a fasted population of children was 8510 L (Osterberg et al., 2006) and 8237 L (Michelet et al., 2016). Osterberg et al. (2006) did not observe a significant effect of BW on V_d_/F. However, Michelet et al. (2016) did describe a significant effect of BW on V_d_/F, suggesting that dose adjustments might be necessary. However, Michelet et al. (2016) also stated that the extent of the covariate effect of BW is quite unclear from the data, since the exponent of the power relation exhibits a quite large uncertainty. When comparing the results of Michelet et al. (2016) and the allometric scaling of the V_d_ (V_d_(human) = V_d_ (pig)^*^(BW human/BW pig)^1^), the piglet is a good model to predict the V_d_ of desmopressin in humans (ratio: 3.88).

The AUC and T_½_ in the different age groups were estimated based on the final pop-PK model. The shrinkage for most important primary PK parameters was acceptable, since the shrinkage of only V_2_/F was higher than 30%. A significant difference between the AUC and T_½el_ of the different age groups was observed. The maximum plasma concentration (C_max_) of the 8-day-old piglets were higher and the T_½el_ was longer in comparison with the other age groups, which could possibly lead to more adverse effects in this age group. When comparing these results with the results obtained by Osterberg et al. (2006) (240 μg; C_max_: ± 40 pg/mL), it can be noted that the C_max_ was much higher in the current study (120 μg; C_max_: ± 250 pg/mL). This can be explained, since this age group was not included in the study of Osterberg et al. (2006). The C_max_ of the other age groups included in the present study are comparable with the C_max_ obtained in the study of Osterberg et al. (2006). The desmopressin concentrations in the 6-month-old age group are lower than the concentrations that are required to obtain antidiuretic effects. It is recommended that in future studies, a 240 μg oral lyophilisate should be administered instead of a 120 μg oral lyophilisate to the 6-month-old piglets.

## Conclusion

The PK parameters of an oral lyophilisate of desmopressin were successfully determined in piglets aged 8 days, 4 weeks, 7 weeks and 6 months using non-linear mixed effects modeling. A two-compartmental model with a dual input function best described the data, since two absorption peaks were observed in the plasma concentration-time curves. Age related differences could be observed in the current study. In human pediatric trials, a one-compartmental model with first-order absorption was fitted to the data and no double peaks were observed in the curves, likely due to the sparse sampling strategy applied in those studies. Therefore, it is recommended that additional studies in children, based on the sampling protocol in the current study, are performed to determine the PK parameters in children based on a full documentation of the plasma concentration-time profile

## Author contributions

EG: conceived the experimental design, performed, and coordinated the animal studies, performed the analyses, drafted the manuscript, and approved the final manuscript as submitted. AV: performed the analyses, drafted the manuscript, and approved the final manuscript as submitted. SC: coordinated the experiments, reviewed and revised the manuscript and approved the final manuscript as submitted. JM: performed the animal studies, reviewed and revised the manuscript and approved the final manuscript as submitted. SS: performed the surgical procedures (anesthesia), reviewed and revised the manuscript and approved the final manuscript as submitted. TvB: performed the surgical procedures (surgery), reviewed and revised the manuscript and approved the final manuscript as submitted. PD: conceived the experimental design, reviewed and revised the manuscript and approved the final manuscript as submitted. JVW: conceived the experimental design, reviewed and revised the manuscript and approved the final manuscript as submitted. MD: coordinated the experiments, reviewed and revised the manuscript and approved the final manuscript as submitted.

### Conflict of interest statement

The authors declare that the research was conducted in the absence of any commercial or financial relationships that could be construed as a potential conflict of interest.

## Abbreviations

ADD: Additive residual error
AIC: Akaike's information criteria
Bio: Fraction passing through depot 1
BSA: Body surface area
BW: Body weight
CL/F: Apparent total body clearance
C_max_: Maximum plasma concentration
CN: Condition number
C_obs_: Observed concentrations
CV: Coefficient of variation
CWRES: Conditional weighted residuals
D_1_: Duration of the zero-order input
EBE: Empirical Bayes estimates
EMA: European medicine agency
F: Absolute oral bioavailability
FDA: Food and drug administration
IIV: Inter-individual variability
IPRED: Individual predicted concentrations
Ka_1_: First-order absorption rate constant
Ka_2_: First-order absorption rate constant
LOQ: Limit of quantification
OFV: Objection function value
PD: Pharmacodynamic
PNE: Primary nocturnal enuresis
PK: Pharmacokinetic
PRED: Population predicted concentrations
Pop-PK: Population pharmacokinetic
Q/F: Apparent intercompartmental flow
RSE: Relative standard error
SE: Standard error
T_lag_: Lag time
V_1_/F: Apparent volume of distribution (central)
V_2_: Vasopressin 2
V_2_/F: Apparent volume of distribution (peripheral)
VPC: Visual predictive check
WRES: Weighted residuals.
