# Supplementary material for: Population Pharmacokinetic Modeling of a Desmopressin Oral Lyophilisate in Growing Piglets as a Model for the Pediatric Population
Source: Front Pharmacol. 2018 Jan 31;9:41. doi: 10.3389/fphar.2018.00041 (PMC5797931; doi:10.3389/fphar.2018.00041)
Supplement: Supplementary file 1 [file Table1.pdf]

Supplementary Table 1

Table 1. Development path of the pharmacokinetic model of desmopressin in growing piglets

Run	OFV	Description	Comments	ΔOFV
1	-158.352	1-compartmental model, first-order absorption	IIV on all parameters, covariance step successful	-
2	-245.768	2-compartmental model, first-order absorption	IIV on all parameters, covariance step not successful	87.4
3	-245.769	2-compartmental model, first-order absorption	IIV on V_2 fixed to 0, covariance step successful, GOF not very good	0.0
4	-227.928	2-compartmental model, first-order absorption	IIV on Q fixed to 0, covariance step successful, GOF good	-17.8
5	-242.160	2-compartmental model, sequential zero-first order absorption	IIV on Q fixed to 0, covariance step successful, GOF good	14.2
6	-279.697	2-compartmental model, dual input	IIV on Q fixed to 0, covariance step not successful	37.5
7	-279.698	2-compartmental model, dual input	IIV on Q, Ka_2, T_{lag} fixed to 0, covariance step successful	0.0
8	-319.418	2-compartmental model, dual input, BW effect on CL	IIV on Q, Ka_2, T_{lag} fixed to 0, covariance step successful	39.7
9	-333.843	2-compartmental model, dual input, BW effect on CL and V_1	IIV on Q, Ka_2, T_{lag} fixed to 0, R matrix	14.4
10	-338.492	2-compartmental model, dual input, BW effect on CL, V_1 and Ka_1	IIV on Q, Ka_2, T_{lag} fixed to 0, covariance step successful	4.6
11	-294.619	2-compartmental model, dual input, BW effect on V_1 and Ka_1	Backward deletion BW effect on CL, significant, covariance step not successful	-43.9
12	-315.999	2-compartmental model, dual input, BW effect on CL and Ka_1	Backward deletion BW effect on V_1, significant, R matrix	21.4
13	-333.843	2-compartmental model, dual input, BW effect on CL and V_1	Backward deletion BW effect on Ka_1, not significant, R matrix	17.8
14	-333.843	2-compartmental model, dual input, BW effect on CL and V_1	IIV on Q, Ka_2, T_{lag} fixed to 0, covariance step successful	0.0
**15**	**-302.068**	**2-compartmental model, dual input, BW effect on Cl and V_1- full dataset**	**IIV on Q, Ka_2, T_{lag} fixed to 0, covariance step successful**	**-31.8**
16	-306.990	2-compartmental model, dual input, BW effect on CL and V_1 – GFR effect on CL	IIV on Q, Ka_2, T_{lag} fixed to 0, T matrix	4.9

OFV: objection function value, IIV: inter-individual variability, V_1/V_2: apparent volume of distribution of the central and peripheral compartment; respectively, Q: apparent intercompartmental flow, T_{lag}: lag time depot 2, GOF: goodness of fit, Ka_1/Ka_2: first-order absorption rate constants from depot 1 and 2; respectively, BW: body weight, CL: clearance, GFR: glomerular filtration rate
